# Supplementary material for: How alginate properties influence in situ internal gelation in crosslinked alginate microcapsules (CLAMs) formed by spray drying
Source: PLoS One. 2021 Feb 25;16(2):e0247171. doi: 10.1371/journal.pone.0247171 (PMC7906420; doi:10.1371/journal.pone.0247171)
Supplement: S1 File — (DOCX) [file pone.0247171.s001.docx]

**Supporting Information**

**How alginate properties influence in situ internal gelation in crosslinked alginate microcapsules (CLAMs) formed by spray drying**

Tina Jeoh ^1¶^*, Dana E. Wong ^1¶#a^, Scott A. Strobel ^1¶#b^, Kevin Hudnall ^1&^, Nadia R. Pereira ^2&^, Kyle A. Williams^3^, Benjamin M. Arbaugh ^1^, Julia C. Cunniffe ^1#c^, and Herbert B. Scher ^1&^

^1^ Department of Biological and Agricultural Engineering, University of California, Davis

Davis, CA 95616, USA

^2^ Laboratory of Food Technology, Universidade Estadual do Norte Fluminense Darcy Ribeiro

Av. Alberto Lamego, 2000, Campos dos Goytacazes, RJ, 28013-602, Brazil

^3^Malvern Panalytical, 117 Flanders Rd, Westborough, MA 01581, USA

^#a^Current Address: DuPont Nutrition & Biosciences, Palo Alto, CA 94304, USA

^#b^Current Address: PivotBio, 2910 Seventh Street, Berkeley, CA 94710, USA

^#c^Current Address: USDA-ARS, Western Regional Research Center, 800 Buchanan Street, Albany, CA 94710, USA

* Corresponding Author

Email: [tjeoh@ucdavis.edu](mailto:tjeoh@ucdavis.edu) (TJ)

^¶^These authors contributed equally to this work

^&^These authors also contributed equally to this work

# Apparent viscosities of commercial alginates


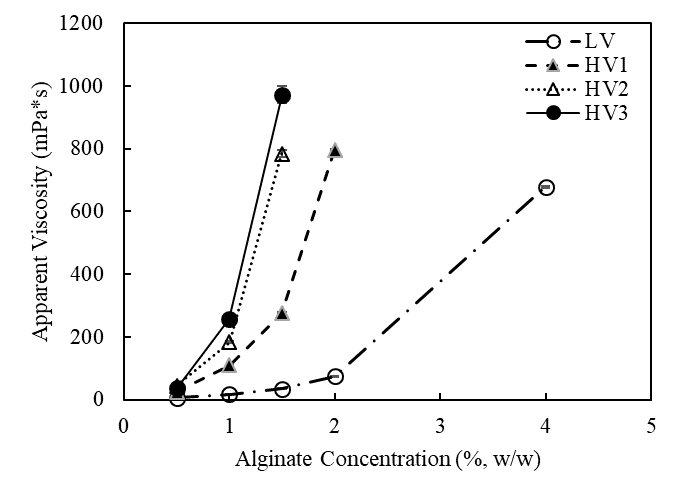
Apparent viscosities of solutions of commercial alginates (Table 1 in main text) at varying concentrations were measured by falling ball viscometry (Fig S. 1). Overall, solution viscosity increased with increasing concentrations of alginate in water. LV alginate, sold as a “low viscosity” alginate by the manufacturer, indeed exhibited the lowest viscosity at all concentrations between 0.5 % and 4 % in water relative to the other alginates. For example, at a concentration of 0.5 %, LV exhibited a viscosity of 6 ± 0.5 mPa*s compared to 45 ± 0.3 mPa*s of HV2, the highest viscosity at this concentration. HV3 had the highest viscosity at a narrow margin over that of HV2 at all concentrations. In fact, viscosity for HV2 and HV3 could only be determined up to 1.5 % because higher concentrations exceeded the range maximum of the falling ball viscometer.

S1 Fig S. 1: Alginate viscosity by source determined by falling ball viscometry. Measurements were performed on 0.5, 1, 1.5, 2, and 4% (w/w) hydrated sodium alginate solutions in water and represent n = 6 over two different days.

# Molecular weight reduction of alginates by partial acid hydrolysis


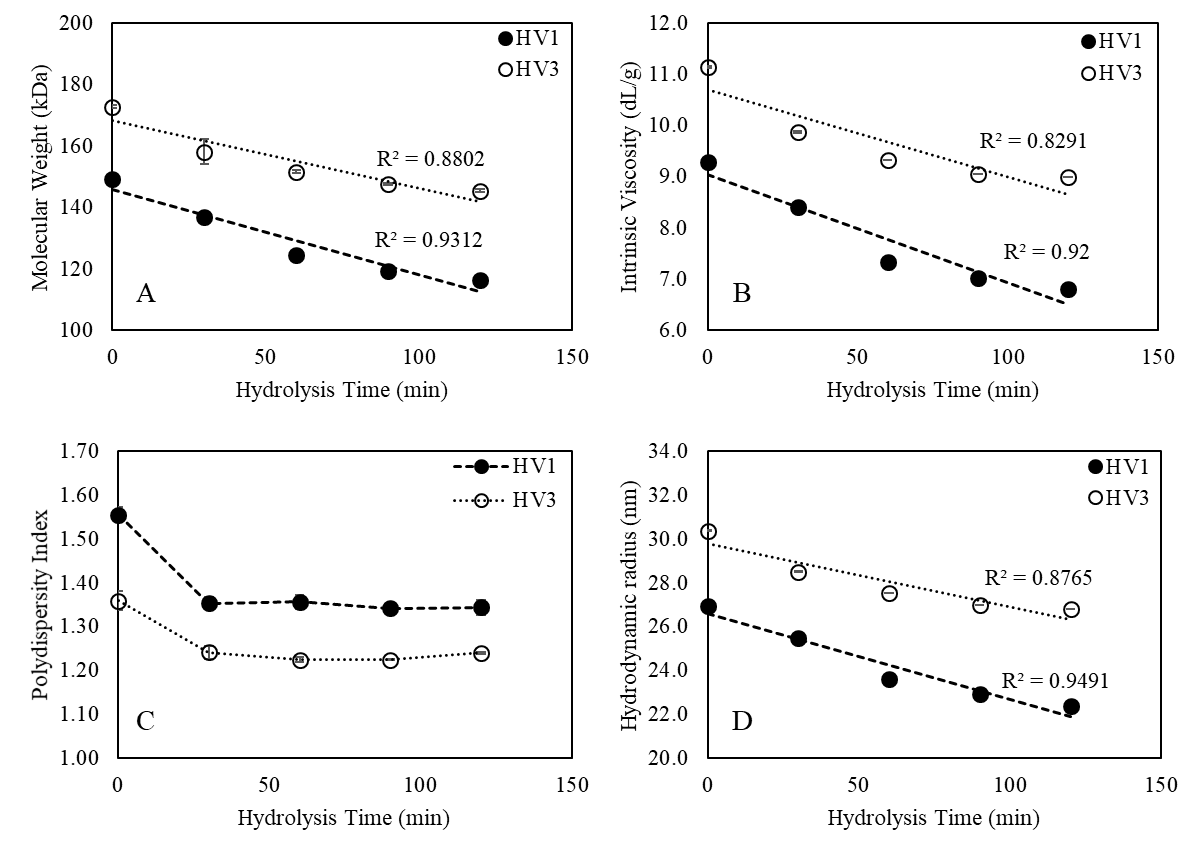
The HV1 and HV3 alginates (10% solutions) were partially hydrolyzed with 2 M sulfuric acid under agitation at room temperature to produce alginates of a range of smaller molecular sizes. The hydrolysis reactions were stopped after 30, 60, 90, and 120 min by neutralization to pH 7 with sodium carbonate. Over the course of 2 hours of acid hydrolysis, the molecular weights of HV1 and HV3 were reduced from 149 to 116 kDa and 172 to 145 kDa, respectively (Fig S. 2A). Consequently, the intrinsic viscosities and hydrodynamic radii of the two alginates were also reduced by acid hydrolysis (Fig S. 2B and D). While the dispersities of the alginates were narrowed by the acid treatment, the majority of the change occurred within the first 30 minutes of the reaction, where dispersities of HV1 and HV3 dropped from 1.6 to 1.3, and 1.4 to 1.2, respectively. The molecular weight analysis from light scattering coupled with refractive index detection accounted for 42 ± 3 % and 38 ± 4 % of the alginates in the sample; i.e. significant fractions of the alginates were not accounted for in the molecular weight analysis, possibly due to degradation in the hydrolysis process.

S1 Fig S. 2: The impact of acid hydrolysis over varying times A) molecular weight, B) intrinsic viscosity, C) dispersity, and D) hydrodynamic radii of two commercial alginates. Lines are drawn the guide the eye. Information on HV1 and HV3 alginates are given in Tables 1 and 2 in the main text. Partially hydrolyzed alginate samples were diluted to 4 mg/mL in 0.05 M sodium sulfate for these analyses.

# Crosslinking in CLAMs formed by partially hydrolyzed alginates

CLAMs were produced from partially hydrolyzed alginates in the same manner as described in the main text using commercial alginates, with a CaHPO_4_ to alginate ratio of 0.125 and feed alginate concentration of 0.5% (w/w). The expectation was that reducing the molecular weights of the alginates would reduce the extents of crosslinking achieved in the CLAMs. Moreover, if molecular weight is the limiting factor in crosslinking, that the reduction would follow a generally linear trend at saturating calcium to alginate ratios as suggested in (Fig 3, main text). Indeed, partial hydrolysis of the alginate samples did reduce crosslinking in the CLAMs (Fig S. 3). However, the extents of crosslinking were reduced from nearly 90 % to ~ 20 %, far exceeding expectations set by trends in Fig 3A (in the main text). As the extent of crosslinking in this study is measured as the insoluble fraction of the CLAMs in water, a possible contribution to the low extents of crosslinking is simply the dissolution of the smaller molecular weight fraction (~ 60%) in both sets of partially hydrolyzed alginate samples. Another possible explanation is the presence of an excess of sodium sulfate in the partially hydrolyzed alginate samples from the neutralization of sulfuric acid that prevented effective calcium crosslinking. In scanning electron micrographs (SEMs), the CLAMs formed with the partially hydrolyzed alginates appeared extensively damaged and hollow, which is highly uncharacteristic of spray dried CLAMs (Strobel et al., 2019, 2016) (Fig S. 4). Despite the possibility of the interference to crosslinking, the CLAMs formed with partially hydrolyzed alginates had ~20 % insoluble fraction.

S1 Fig S. 3: The extents of crosslinking achieved in (0.125 calcium to alginate) CLAMs using partially hydrolyzed alginates plotted with respect to the molecular weights. Lines were drawn to guide the eye. Linear fit to the extent of crosslinking as a function of molecular weight for 0.125 CaHPO_4_/alginate ratio CLAMs in Fig 3A of the main text is also shown here (solid line).


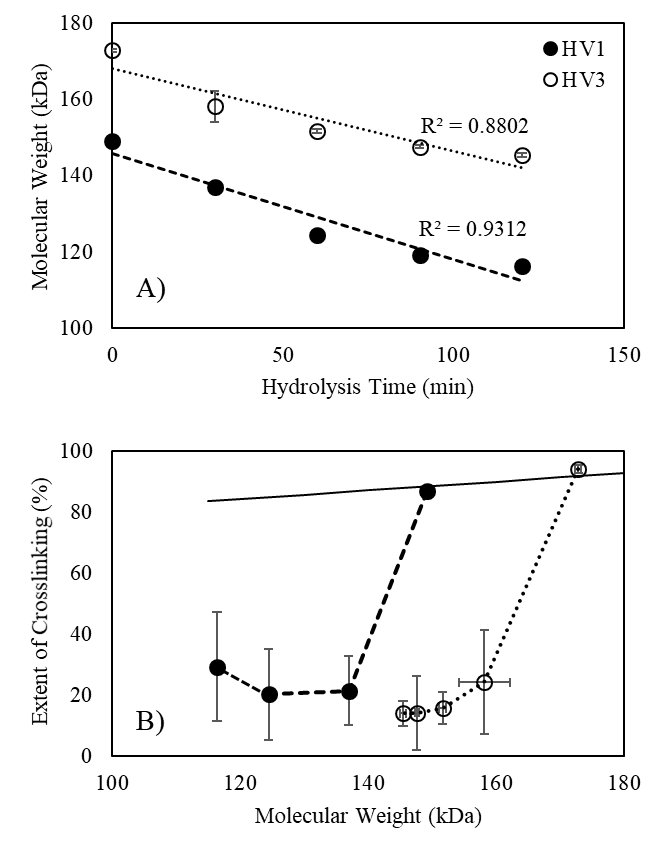


# SEMs of CLAMs formed using partially hydrolyzed HV1 and HV3 alginates

CLAMs have previously been described as having a ‘deflated ball’ or ‘bowl’ shaped morphology when formed without cargo (Strobel et al., 2016, 2019). The bowl shape of empty CLAMs is hypothesized to be due to rapid skin formation at the droplet interface, followed by collapse of the skin as internal moisture evaporates. Typical spray dried CLAMs have neither exhibited blowholes, nor appeared to be brittle. In contrast, CLAMs produced from the partially hydrolyzed alginates generated in this study appeared as hollow structures exhibiting significant fracturing (Fig S. 4).


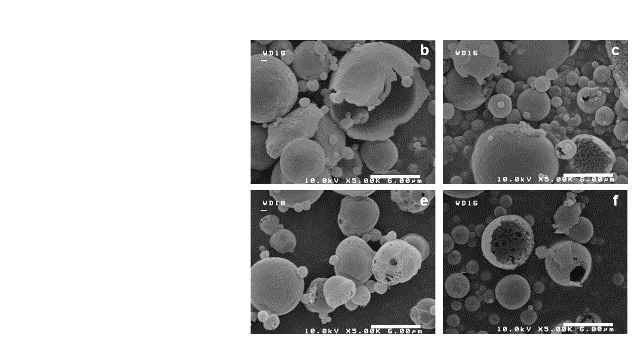


(a)

(b)

(c)

(d)

S1 Fig S. 4: Scanning electron micrographs of CLAMs made with partially hydrolyzed HV1 (a and b) and HV3 (c and d). All CLAMs were made from feed solutions of 0.5% alginate and 0.125 calcium/alginate. Samples were coated with 15 nm of gold (Cressington 108 Auto Coating System, Watford, UK) and imaged at 10 kV using a Hitachi S-4100T FE-SEM (Krefeld, Germany).

# Comparing the FTIR spectra of CLAMs, succinic acid and CaHPO_4_

The FTIR spectra of CLAMs exhibit a peak centered at 1710 cm^-1^ suggesting the presence of alginic acids in the sample. The CLAMs formulation contains succinic acid that also has a carboxylic acid functionality. To confirm that the peak assigned to alginic acid in CLAMs is not due to the presence of succinic acid, the FTIR spectra for the succinic acid used in the formulation was collected and overlaid (Fig S. 5). The carbonyl peak associated with succinic acid absorbs maximally at 1685 cm^-1^ and is offset from that of the carbonyl peak of the CLAMs sample. Moreover, the relative magnitude of the peaks in the succinic acid spectra compared to those of the CLAMs spectra suggest that the succinic acid contribution to the overall CLAMs spectra is small. We thus conclude that the 1710 cm^-1^ absorption peak in the CLAMs spectra is unique to CLAMs and not a contribution of the succinic acid in the formulation. The FTIR spectra of CaHPO_4_ is also overlaid in Fig S. 5 for reference.


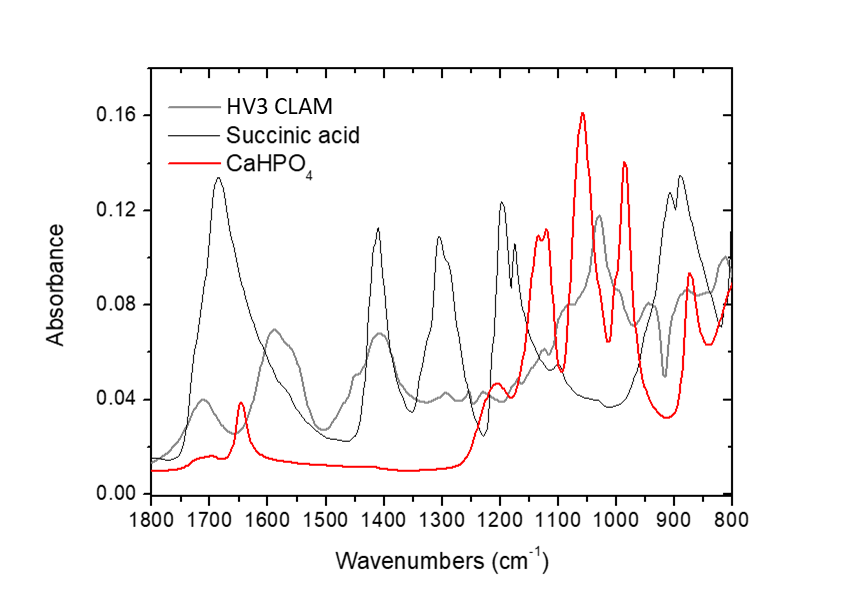


S1 Fig S. 5: Overlay of FTIR spectra of HV3 CLAMs, succinic acid and CaHPO_4_.

# Select SEMs of CLAMs

SEM images of select CLAMs are shown in Fig S. 6. SEMs of CLAMs formed with varying calcium to alginate ratios using LV alginates can be seen as Fig 3 in (Strobel et al., 2019).


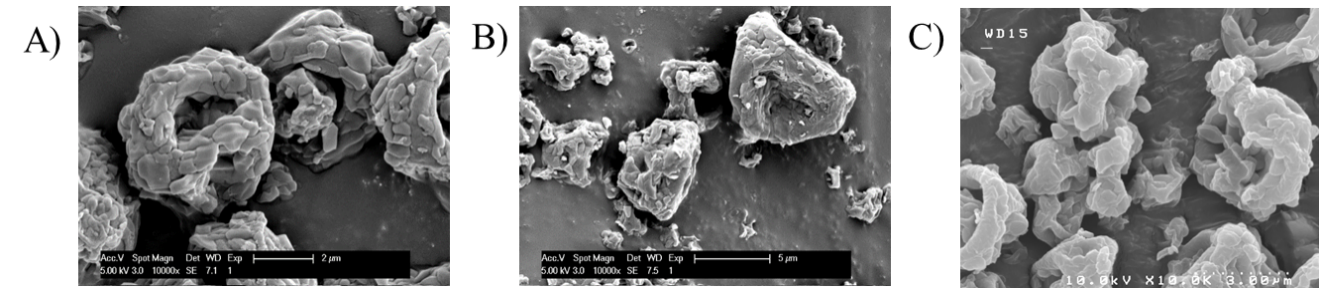


S1 Fig S. 6: SEMs of CLAMs formed with 0.125 CaHPO_4_:Alginate using A) HV1, scale bar = 2 µm, B) HV2, scale bar = 5 µm, and C) HV3, scale bar = 3 µm.

# References

Strobel, S.A., Scher, H.B., Nitin, N., Jeoh, T., 2019. Control of physicochemical and cargo release properties of cross-linked alginate microcapsules formed by spray-drying. J. Drug Delivery Sci. Technol. 49, 440–447. https://doi.org/10.1016/j.jddst.2018.12.011

Strobel, S.A., Scher, H.B., Nitin, N., Jeoh, T., 2016. In situ cross-linking of alginate during spray-drying to microencapsulate lipids in powder. Food Hydrocolloids 58, 141–149. http://dx.doi.org/10.1016/j.foodhyd.2016.02.031
