# Supplementary material for: How alginate properties influence in situ internal gelation in crosslinked alginate microcapsules (CLAMs) formed by spray drying
Source: PLoS One. 2021 Feb 25;16(2):e0247171. doi: 10.1371/journal.pone.0247171 (PMC7906420; doi:10.1371/journal.pone.0247171)
Supplement: Attachment — Submitted filename: PONE-D-29628.docx [file pone.0247171.s002.docx]

Dear Editor,

Thank you for giving me opportunity for review this manuscript.

This manuscript focused on crosslinking of alginates. It is well written and results are explained well and supported by suitable literature. The research question is clearly mentioned. I found few points need to be add which improve the article. For instance

Line 91: What are others methods of crosslinking other than spray drying, kindly mention that

Line 150: Kindly mention reference for this method if authors have developed the method then kindly mention that

Line 187: What is basis for selection of inlet temperature? Does inlet temperature affect the alginate crosslinking?

Line 188: What is outlet temperature? Does outlet temperature affect the alginate crosslinking?

Line 197: Kindly give reference for the method

Line 225: What is yield during spray if any losses are there kindly mention in manuscript

Line 236: Authors have taken calcium phosphate for this study, why calcium phosphate has been considered? Does salt type influence the crosslinking?

Line 249: There is any method to get 100 % crosslinking

Line 256: What are other properties of alginate which may influence the crosslinking

Line 367: What is moisture content of CLAMs does moisture content of CLAMs affect the crosslinking

Line 378: kindly mention the limitations of this study if any
