# Supplementary material for: How alginate properties influence in situ internal gelation in crosslinked alginate microcapsules (CLAMs) formed by spray drying
Source: PLoS One. 2021 Feb 25;16(2):e0247171. doi: 10.1371/journal.pone.0247171 (PMC7906420; doi:10.1371/journal.pone.0247171)
Supplement: Attachment — Submitted filename: PLOS ONE-review.docx [file pone.0247171.s003.docx]

The authors studied about “How alginate properties influence in situ internal gelation in Crosslinked Alginate Microcapsules (CLAMs) formed by spray drying”. This study is performed alternative to external gelation to prove the advantage of this study. This represented study has to clarify the following points before acceptance in this journal:

1. The microcapsule preparation method used calcium as crosslinker, the authors should aware the used calcium concentration should not exceed physiological intake limit. Excessive calcium consumption will cause severe health complication specially cardiac complications. Hence, explain in the manuscript how much quantity of calcium used and that quantity are in acceptable limit or not.
2. Ammonium hydroxide is used as a base in this study to maintain higher pH. It is boiling point almost near to room temperature, so it can evaporate in room atmosphere before spray or it can completely evaporate in nozzle head. In this concern how ammonium hydroxide based pH theory works in microcapsule preparation. Please explain.
3. In page no:11, line no: 226 mentioned that crosslinked alginate microencapsulation. What type of molecule is encapsulated into the microcapsule?, like that in graphical abstract mentioned dry cargo-loaded CLAM. What type of cargo is loaded into the CLAM?
4. Extend of crosslinking data of external crosslinked microparticles is not mentioned in the manuscript and it is not compared with CLAM. Please include that in the manuscript.
