# Supplementary material for: How alginate properties influence in situ internal gelation in crosslinked alginate microcapsules (CLAMs) formed by spray drying
Source: PLoS One. 2021 Feb 25;16(2):e0247171. doi: 10.1371/journal.pone.0247171 (PMC7906420; doi:10.1371/journal.pone.0247171)
Supplement: Attachment — Submitted filename: 201216 PONE-D-29628 Response to Reviewers.docx [file pone.0247171.s004.docx]

PONE-D-20-29628 Response to Reviewers

How alginate properties influence in situ internal gelation in Crosslinked Alginate Microcapsules (CLAMs) formed by spray drying

PLOS ONE

Note: page and line numbers of the revised manuscript are referenced in our response.

Reviewer 1

Thank you for giving me opportunity for review this manuscript.

This manuscript focused on crosslinking of alginates. It is well written and results are explained well and supported by suitable literature. The research question is clearly mentioned.

*Thank you to the reviewer for their kind assessment of our work.*

I found few points need to be add which improve the article. For instance

Line 91: What are others methods of crosslinking other than spray drying, kindly mention that

*Line 72 (formally line 91) mentions both internal and external gelation approaches for crosslinking alginate which serve as categorical descriptions of crosslinking methods. We have added the following reference to a review of alginate microencapsulation, which describes the internal and external gelation methods in greater detail.*

*Reis CP, Neufeld RJ, Vilela S, Ribeiro AJ, Veiga F. Review and current status of emulsion/dispersion technology using an internal gelation process for the design of alginate particles. J Microencapsul [Internet]. 2006 Jan [cited 2020 Dec 9];23(3):245–57. Available from: http://www.tandfonline.com/doi/full/10.1080/02652040500286086*

Line 150: Kindly mention reference for this method if authors have developed the method then kindly mention that

*The methodology for HPLC detection of PMP-labeled carbohydrates was derived from the works of Honda et al. (1989) and Wang et al. (2015). These references were included in the next paragraph.*

Line 187: What is basis for selection of inlet temperature? Does inlet temperature affect the alginate crosslinking?

*In this study, the spray dryer inlet temperature was maintained at 150°C, for the sake of consistency with our previous studies. The selection of inlet temperature could certainly affect the extent of alginate crosslinking: a higher inlet temperature could vaporize ammonia and solubilize calcium more rapidly, resulting in more extensive crosslinking. We maintained constant spray drying inlet temperature to focus on alginate properties influencing gelation in these studies.*

Line 188: What is outlet temperature? Does outlet temperature affect the alginate crosslinking?

*The temperature at the outlet of the spray dryer is dependent on a variety of factors and spray parameters including the inlet temperature, liquid feed flow rate, drying air flow rate, drying air humidity, etc. As such, the outlet temperature can vary from run to run. For the operational parameters specified, the outlet temperature typically ranged from 85 – 95°C. For the CLAMs process, outlet temperature is not thought to affect alginate crosslinking. The manuscript has been edited to include the outlet temperature range in the methods section.*

Line 197: Kindly give reference for the method

*We have supplied reference 10.*

Line 225: What is yield during spray if any losses are there kindly mention in manuscript

*Process yields for benchtop spray dryers such as a Buchi B290 are generally far lower than what can be achieved at industrial scale. As such, any reported yields would be of little industrial relevance.*

Line 236: Authors have taken calcium phosphate for this study, why calcium phosphate has been considered? Does salt type influence the crosslinking?

*For consistency with our previous studies, calcium phosphate was utilized as the acid-soluble calcium salt in this work. Previous researchers have explored utilizing several other acid-soluble insoluble calcium salts (e.g. calcium carbonate) to crosslink alginate by emulsification / internal gelation, and the salt type (and particle size) did indeed affect the properties of the resulting alginate beads (Reis et. al 2006). A recently published study by our group utilizes chelated calcium chloride (Wong 2020) which serves as an example of effective use of an alternative calcium salt for alginate crosslinking.*

Line 249: There is any method to get 100 % crosslinking

*Ion mediated alginate crosslinking is reversible by its very nature. Using the crosslinking metric in this study, 100% crosslinking is theoretically possible. This study was exploring the question of what alginate properties contribute to (or prevent) achieving this.*

Line 256: What are other properties of alginate which may influence the crosslinking

*We kindly point the reviewer to lines 237-239: “Average polymer size, composition, and viscosity have all been demonstrated to impact the ability of alginates to gel in the presence of multivalent ions.”*

Line 367: What is moisture content of CLAMs does moisture content of CLAMs affect the crosslinking

*As benchtop spray drying produces limited quantities of dry material, and moisture content measurement is a destructive measurement, the moisture content was not routinely measured as part of this study. In one of our previous studies (Strobel et al 2019), we found that the moisture content of CLAMs (approx 6-7%) was not significantly influenced by the calcium content (extent of crosslinking).*

*Strobel, S. A.; Allen, K.; Roberts, C.; Jimenez, D.; Scher, H. B.; Jeoh, T. Industrially-Scalable Microencapsulation of Plant Beneficial Bacteria in Dry Cross-Linked Alginate Matrix. Ind. Biotechnol.* ***2018****, 14 (3), 138–147.*

Line 378: kindly mention the limitations of this study if any

*We welcome specific suggestions for limitations to include in such a discussion.*

Reviewer 2

The authors studied about “How alginate properties influence in situ internal gelation in Crosslinked Alginate Microcapsules (CLAMs) formed by spray drying”. This study is performed alternative to external gelation to prove the advantage of this study. This represented study has to clarify the following points before acceptance in this journal:

1. The microcapsule preparation method used calcium as crosslinker, the authors should aware the used calcium concentration should not exceed physiological intake limit. Excessive calcium consumption will cause severe health complication specially cardiac complications. Hence, explain in the manuscript how much quantity of calcium used and that quantity are in acceptable limit or not.

*Thank you for this feedback. Our present work describes properties of CLAMs outside the context of any specific application. We certainly understand that calcium intake limit could be relevant for pharmaceutical or food applications. The NIH recommends an upper limit of 2500 mg calcium per day for adults aged 19-50. Our conservative estimate, using the highest calcium content in Figure 1 (empty CLAMs prepared with Ca/A = 0.25) is that CLAMs would contain no more than 4.2% Ca by weight. Based on these estimates, an adult could consume 59 g CLAMs without exceeding the NIH threshold. Should an application require higher intake of CLAMs, the authors would suggest using a lower Ca/A ratio or exploring alternative acid-soluble salts of divalent cations.*

*We would like to re-emphasize, however, that end-use of CLAMs can be unrelated to human intake. For example, as mentioned in the first paragraph of the introduction, CLAMs can be used to confer protection to plant beneficial bacteria used in agricultural applications. Other example applications include enzyme encapsulation for homecare (e.g. cleaning) products.*

2. *Ammonium hydroxide is use*d as a base in this study to maintain higher pH. It is boiling point almost near to room temperature, so it can evaporate in room atmosphere before spray or it can completely evaporate in nozzle head. In this concern how ammonium hydroxide based pH theory works in microcapsule preparation. Please explain.

*Pure ammonium hydroxide does indeed have a high vapor pressure at room temperature. In CLAMs formulation, ammonium hydroxide is combined with succinic acid, forming ammonium succinate, which does not vaporize substantially prior to spray drying. However, during the spray drying process, interconversion of ammonium ion to ammonia vapor results in a pH drop that solubilizes the calcium salt and initiates alginate crosslinking.*

3. In page no:11, line no: 226 mentioned that crosslinked alginate microencapsulation. What type of molecule is encapsulated into the microcapsule?, like that in graphical abstract mentioned dry cargo-loaded CLAM. What type of cargo is loaded into the CLAM?

*In this work, CLAMs were prepared empty (with no specific cargo/payload) to facilitate the study of the spray dried crosslinked alginate matrix. A generic cargo is included in the graphical abstract to call out that cargo would be included in the spray dryer feed when preparing microcapsules. Some examples are given in the first paragraph of the introduction.*

4. Extend of crosslinking data of external crosslinked microparticles is not mentioned in the manuscript and it is not compared with CLAM. Please include that in the manuscript.

*The section titled “The role of composition on crosslinking in CLAMs” with the accompanying Figure 4 provides data on crosslinking of external crosslinking microparticles. Figure 4A is a comparison of external gelation and CLAMs.*

Reviewer 3

The paper entitled “Alginate properties impacting gelation in spray-dried crosslinked alginate microcapsules” is related to influence of alginate molecular properties and their effect on extend of cross linking in in-situ gelation in CLAMs produced by spray drying technique. The approach of the manuscript is interesting and could provide information. The paper is well written and methodology is understandable.

*Thank you to the reviewer for the kind assessment of our work.*

I would like to suggest the Editor to accept the manuscript with minor corrections.

1. Materials and methods require details in explaining the statistical analysis.

*A section was added to describe the statistical analyses conducted in this study.*

2. In table 2, authors explain significant difference only for G/M ratio. But they didn’t say anything about significant difference for other parameters in the table. Are there? Also please use same number of decimals for all values.

*Coefficients denoting statistical differences have now been added to all the data in Table 2 for which direct comparisons are relevant. The significant figures shown in the table best reflect the accuracy of the variety of methods used to determine the tabulated values.*

3. Figure 5 caption explains the same as in discussion part. Please change it.

*The figure caption is descriptive of the peaks in the figure, while the discussion elaborates on the context of why these peaks occur. Upon reviewing the Figure 5 caption, we feel that if we remove any information from the caption, the figure would not stand on its own. The caption is not a replication of the discussion and conveys accurate information. Thus, we respectfully disagree with the reviewer and choose to retain the original caption.*

*Note to the editor*

*We are open to reasonable compromise if the editor requires.*

Reviewer 4

Review Comments Manuscript Title: How alginate properties influence in situ internal gelation in Crosslinked Alginate Microcapsules (CLAMs) formed by spray drying The following aspects may be considered to improve the work.

1. Improve quality of all figures. Some values are just not readable!

*The figure quality are now all according to the journal’s standards (and have been processed using the PACE tool suggested by the journal).*

2. Elaborate the underlying chemical changes as equations.

*The crosslinking reported in this study are physical (electrostatic) crosslinks and not chemical crosslinks.*

3. Improve discussion with recent (2019-20) articles on spray drying + crosslinking (perhaps even other 7 molecules).

*As our study focuses on alginate as the encapsulation matrix, we intentionally focused our discussion on alginates. The unique aspect of the CLAMs technology is that it facilitates physical, electrostatic crosslinking of alginates by divalent calcium cations. We would like to emphasize that this has not been achieved directly achieved by spray drying in any other processes.*

4. Add details on the purity and composition of all raw materials. How would they impact the findings of this study?

*The alginates used and discussed in this work were characterized physically and chemically, and were summarized in Table 2. The commercially sourced alginates were used as received to show effective crosslinking is achievable without the need for pre-processing (which would add cost and time to an industrially scaled system). Unfortunately, suppliers do not provide purity or composition information to customers.*

5. Add details on the drying mechanism in the spray dryer (relevant to the microcapsule formation).

*We have added the following sentence in the introduction that references our work on modeling the mechanism of particle formation and drying in the spray dryer for microcapsule formation:*

*“The mechanisms of particle formation and drying in the spray dryer is well-documented and recently modeled specifically for the CLAMs process to show exponential moisture removal during drying (21).”*

6. What about the post-encapsulation quality?

*We would like to call the reviewer’s attention to our previous work where we extensively assess the quality and properties of the CLAMs as an encapsulation strategy* ^1–6^ *(these are also included in this manuscript, referenced from the first paragraph of the introduction). This work specifically focuses on the role of alginate properties that influence gelation in CLAMs. We had seen in our suite of previous work that controlling gelation can be important to controlling quality.*

(1)	Strobel, S. A.; Scher, H. B.; Nitin, N.; Jeoh, T. In Situ Cross-Linking of Alginate during Spray-Drying to Microencapsulate Lipids in Powder. *Food Hydrocoll.* **2016**, *58*, 141–149. http://dx.doi.org/10.1016/j.foodhyd.2016.02.031.

(2)	Santa-Maria, M.; Scher, H.; Jeoh, T. Microencapsulation of Bioactives in Cross-Linked Alginate Matrices by Spray-Drying. *J. Microencapsul.* **2012**, *29* (3), 286–295. https://doi.org/10.3109/02652048.2011.651494.

(3)	Strobel, S. A.; Hudnall, K.; Arbaugh, B.; Cunniffe, J. C.; Scher, H. B.; Jeoh, T. Stability of Fish Oil in Calcium Alginate Microcapsules Cross-Linked by In Situ Internal Gelation During Spray Drying. *Food Bioprocess Technol.* **2020**, *13*, 275–287. https://doi.org/10.1007/s11947-019-02391-y.

(4)	Strobel, S. A.; Allen, K.; Roberts, C.; Jimenez, D.; Scher, H. B.; Jeoh, T. Industrially-Scalable Microencapsulation of Plant Beneficial Bacteria in Dry Cross-Linked Alginate Matrix. *Ind. Biotechnol.* **2018**, *14* (3), 138–147.

(5)	Strobel, S. A.; Scher, H. B.; Nitin, N.; Jeoh, T. Control of Physicochemical and Cargo Release Properties of Cross-Linked Alginate Microcapsules Formed by Spray-Drying. *J. Drug Deliv. Sci. Technol.* **2019**, *49*, 440–447. https://doi.org/10.1016/j.jddst.2018.12.011.

(6)	Wong, D. E.; Cunniffe, J. C.; Scher, H. B.; Jeoh, T. Chelator Regulation of In Situ Calcium Availability to Enable Spray- Dry Microencapsulation in Cross-Linked Alginates. *ACS Omega* **2020**, *5*, 24453–24460.
