# Supplementary material for: How alginate properties influence in situ internal gelation in crosslinked alginate microcapsules (CLAMs) formed by spray drying
Source: PLoS One. 2021 Feb 25;16(2):e0247171. doi: 10.1371/journal.pone.0247171 (PMC7906420; doi:10.1371/journal.pone.0247171)
Supplement: Attachment — Submitted filename: 210124 PONE-D-296281R1 Response to Reviewers.docx [file pone.0247171.s005.docx]

Reviewer #2: (No Response)

Reviewer #5: (No Response)

Reviewer #6: The authors did address all the comments by the reviewers sufficiently. I agree with the author that the comment by reviewer 3 regarding the caption of Figure 5 is not applicable since the Figure is very complex and an extensive caption to explain is necessary.

Thank you for the feedback!

Reviewer #7: This manuscript reported that “How alginate properties influence in situ internal gelation in crosslinked alginate microcapsules(CLAMs) formed by spray drying”. The authors compared the effects of alginic acid molecular weight, size distribution and molecular composition on CLAMs gelation. Overall, the manuscript was well written, experiments were well designed, the data interpretation is sound and the conclusion drawn by authors is firmly supported by the data provided. Therefore, it would be suitable for publication in the journal "PLOS ONE" without further revision.

Thank you for your time to review our work.

Reviewer #8: This work studies the impact of alginate properties in crosslinked alginate microcapsules formed by CLAMs process.

The authors described various factors that affect the extent of crosslinking, with a particular focus on the influence of commercially available alginates. Due to lack of information of commercial alginates, the authors performed a characterisation of these to study the impact of their properties on the extent of crosslinking in CLAMs. Interestingly, the impact of alginate properties is dependent on the method utilised for crosslinking, where spray-dried CLAMs are mainly influenced by the molecular weight or density of alginates at saturating salt concentrations. The paper is well written and the materials and methods are clear and provide sufficient information. This manuscript has been already reviewed and most of my doubts have been solved during this round of revision. I have only one question regarding the G/M ratio. The authors described that by external gelation, 5% and 93% of crosslinking is achieved with PolyMs and PolyGs, respectively. On the other hand, 50% crosslinking was observed with PolyMs and PolyGs by CLAMs process, which contradicts the expectations of the authors. I believe these results need to be further discussed by the authors. Is this equal crosslinking of polyMs and polyGs influenced by alginic acid? are there any other parameters that may influence the outcomes observed?.

Thank you to the reviewer for this feedback. Indeed the high ‘crosslinking extents’, or more accurately ‘gelation extents’ observed in the PolyM formulations was surprising. As we describe in our manuscript, there is extensive documentation in the literature showing that PolyM regions of alginates do not effectively form calcium-mediated crosslinks. When we looked into this further by FTIR, we found indication of alginic acid gelation in the CLAMs. In addition to ion-mediated crosslinking, a second common mechanism for alginate gelation is to form ‘acid gels’ stabilized by intermolecular hydrogen-bonds. It is very possible that the CLAMs process facilitates localized alginic acid gelation within the particles as drying and pH drop occurring rapidly and simultaneously could cause localized ‘hot spots’ of concentrated protons. We have elaborated further on p. 16 of the manuscript.

Reviewer #9: The authors have adequately responded to all reviewer comments. The paper is well-written, and will be an important contribution to this area of pharmaceutical development, particle engineering and spray drying.

Thank you for your kind assessment.

Reviewer #10: The authors present preparation of crosslinked alginate microcapsules (CLAMs) with alginates of different properties using spray drying technique. The study seems to have been designed well and the experiments are done rigorously. The authors seem to have addressed most of earlier concerns raised be reviewers. However, there are few concerns that need to be fixed before it is finalized. Specific comments: In the process of preparation of the CLAMs the feed suspension contains calcium hydrogen phosphate, succinic acid, and ammonium hydroxide along with Alginates. When the crosslinking takes place by calcium ions due to pH change, the crosslinking possibilities are; it can crosslink the alginates directly (alginate-Ca-alginate), or it can crosslink alginate through a succinate-bridge (alginate-Ca-succinate-Ca-alginate), or it can conjugate an alginate to one succinate carboxyl group and the other remains as carboxylic acid, or it can be a mixture of these. The COO- groups in succinates can exist in ionic form of COO- NH4+ (in presence of NH3 and H2O or NH4OH), and COO- H3O+ (on evaporation of NH3), and in equilibrium with COOH. The mono carboxylic acid of the succinate can also be detectable at ~1710 cm-1 in the FTIR along with alginic acid if present. To confirm if the acid carbonyl observed is due to alginic or succinic or both, the process should be tested with and an alkyl acid containing only one carboxylic acid instead of a dicarboxylic acid in the process. Otherwise, the formation of alginic acid should be confirmed with another method of analysis to eliminate other possibilities.

We thank the reviewer for these thoughtful comments. We have indeed ruminated over these crosslinking possibilities and here are our thoughts on the possibilities raised by the reviewer:

(alginate-Ca-alginate): these are assigned to Peaks 2 and 4 (1595 cm^-1^ and 1410 cm^-1^) in Fig. 5 as explained in the manuscript.

(alginate-Ca-succinate-Ca-alginate): our expectation is that this variation that includes a succinate bridge would weaken the alginate-Ca association and thus manifest as a ‘red-shift’ of Peaks 2 and 4 (Fig. 5); i.e. relative to the corresponding peaks in sodium-alginate, we would see these peaks shift to a slightly lower wavenumber indicating a weakened alginate-Ca bond. We do not observe either an increase or decrease in wavenumber of these characteristic peaks. Thus we conclude that if the succinate bridge exists, it does not interfere with the alginate-Ca bond interactions, or that the bridging is not sufficiently significant to manifest as an additional peak or a shift in the main peak.

Other possible ion-mediated bridging alternatives would have to be considered similarly, asking the question of the influence on Peak 2 and 4. Considering that Peaks 2 and 4 are dominant in the spectra of the CLAMs and sodium alginate samples, we conclude that a COO-(cation) interaction persists in the CLAMs.

The reviewer points out that there can also be amine salts formed. We do indeed see a peak corresponding to the formation of an amine salt as Peak 3 (1550 cm^-1^) in the CLAMs spectra (noted in the figure caption). What role this plays in gelation is not clear just based on the FTIR spectra.

As the reviewer points out, the mono carboxylic acids of succinic acid also absorbs strongly around 1700 cm^-1^. It turns out that the carboxylic acid peak of succinic acid is at 1685 cm^-1^, which is not observable in the CLAMs spectra where the peak assigned to carboxylic acids of alginic acid is at 1710 cm^-1^ (Figure S.5 in supplementary information). We posit that through the CLAMs process, ammonium succinate is formed (possibly corresponding to the 1550 cm^-1^ peak).

At this stage in our work, we do feel that we have presented sufficient evidence to support the contribution of alginic acid to gelation in crosslinked alginate particles formed by the CLAMs process. We present evidence showing little to no gelation of PolyM alginates in the conventional external gelation method compared to 50% gelation by the CLAMs process. We also present evidence for formation of alginic acids in the FTIR spectra. Taken together, we speculate that the CLAMs process facilitates gelation mechanisms in addition to calcium-mediated crosslinking, and that alginic acid gelation is a strong candidate for this additional mechanism. It is possible that other mechanisms contribute as well and we agree that further experiments and analyses would provide further insights. Unfortunately, we are not in a position at this time to conduct further experiments and analyses within a reasonable period. We are confident in the evidence that we do provide and feel that with this work, we contribute new insights to alginate-based microencapsulation. We hope that the reviewer and editor will agree with us.

Reviewer #11: The authors present preparation of crosslinked alginate microcapsules (CLAMs) with alginates of different properties using spray drying technique. The study seems to have been designed well and the experiments are done rigorously. The authors seem to have addressed most of earlier concerns raised be reviewers. However, there are few concerns that need to be fixed before it is finalized. Specific comments: In the process of preparation of the CLAMs the feed suspension contains calcium hydrogen phosphate, succinic acid, and ammonium hydroxide along with Alginates. When the crosslinking takes place by calcium ions due to pH change, the crosslinking possibilities are; it can crosslink the alginates directly (alginate-Ca-alginate), or it can crosslink alginate through a succinate-bridge (alginate-Ca-succinate-Ca-alginate), or it can conjugate an alginate to one succinate carboxyl group and the other remains as carboxylic acid, or it can be a mixture of these. The COO- groups in succinates can exist in ionic form of COO- NH4+ (in presence of NH3 and H2O or NH4OH), and COO- H3O+ (on evaporation of NH3), and in equilibrium with COOH. The mono carboxylic acid of the succinate can also be detectable at ~1710 cm-1 in the FTIR along with alginic acid if present. To confirm if the acid carbonyl observed is due to alginic or succinic or both, the process should be tested with and an alkyl acid containing only one carboxylic acid instead of a dicarboxylic acid in the process. Otherwise, the formation of alginic acid should be confirmed with another method of analysis to eliminate other possibilities.

Reviewer 11 seems to be a duplicate of Reviewer 10. Please see our response to Reviewer 10.
